# Supplementary material for: K–8 Classroom Self-Collection Using XpressCollect Nasal Swab: A Usability and Efficacy Study
Source: Diagnostics (Basel). 2022 May 17;12(5):1245. doi: 10.3390/diagnostics12051245 (PMC9140182; doi:10.3390/diagnostics12051245)
Supplement: Supplementary file 1 [file diagnostics-12-01245-s001.zip › Figure S2.pdf]

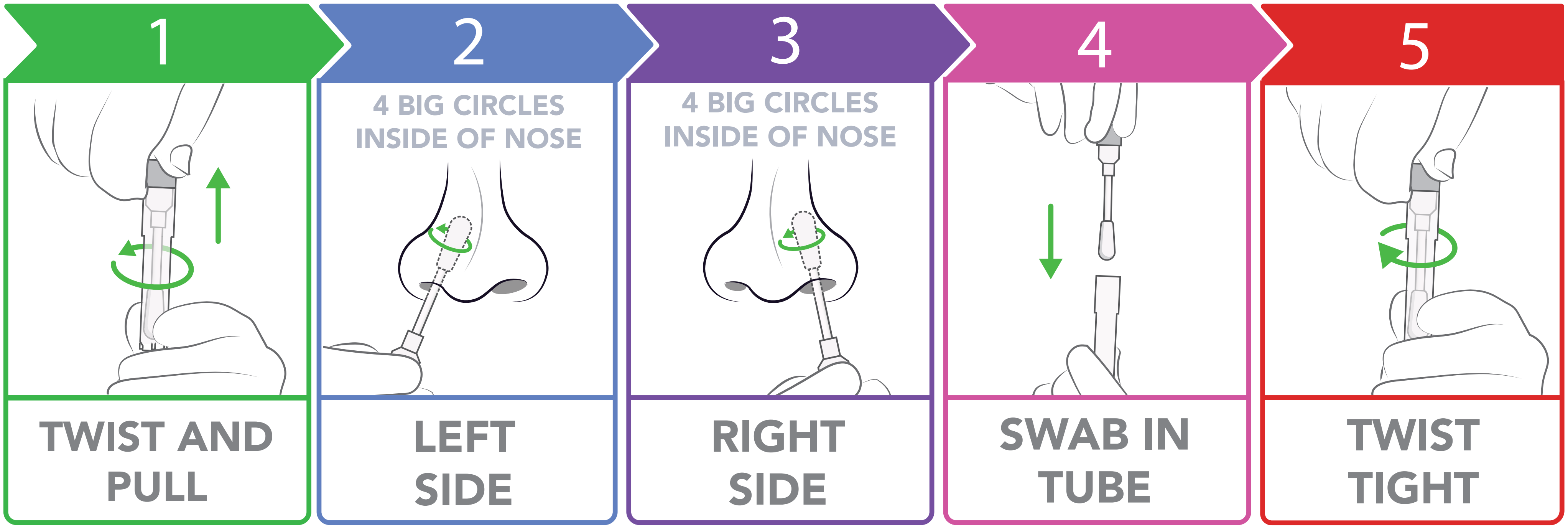
1
TWIST AND PULL
2
4 BIG CIRCLES INSIDE OF NOSE
LEFT SIDE
3
4 BIG CIRCLES INSIDE OF NOSE
RIGHT SIDE
4
SWAB IN TUBE
5
TWIST TIGHT
