# Supplementary material for: K–8 Classroom Self-Collection Using XpressCollect Nasal Swab: A Usability and Efficacy Study
Source: Diagnostics (Basel). 2022 May 17;12(5):1245. doi: 10.3390/diagnostics12051245 (PMC9140182; doi:10.3390/diagnostics12051245)
Supplement: Supplementary file 1 [file diagnostics-12-01245-s001.zip › File S1.pdf]

Classroom Testing Introduction

The goal of classroom testing is to provide another safety measure for teachers, students, staff, and families involved with in-person education.

The Xpress Collect kit is intended to be used by students in grades K-8 to self-collect nasal samples to be sent to a laboratory for respiratory illness testing to prevent disease spread. The Xpress Collect kit contains general purpose laboratory equipment to facilitate the unsupervised self-collection of nasal swab specimens.

Kit Contents:

- Teacher Guide
- Packaged Sample Tubes (Each package includes 1 swab inside a tube)
- Student instructions
- Web link for Instruction Video

Materials Needed Not Included in this Kit:

- Tissues
- Hand Sanitizer or access to sink for students to clean hands

It is recommended that you try Xpress Collect on yourself prior to administering a classroom sample collection. This can inform you on how to use the product on yourself, assure comfort with the product and be prepared for questions students may have. There are a few key safety items to recognize prior to beginning classroom sample collection. These include the following:

- Students must insert the product until the natural stopping point in the nose.
  - IT SHOULD NOT BE INSERTED ALL THE WAY and should not cause discomfort
- Students should be encouraged to self-collect.
  - Where possible, AVOID helping students. Helping students self-collect is not time efficient and may increase disease spread.
- Students should be encouraged to place sample collecting swab back into the tube without touching another surface.
  - Once a sample is collected, students should avoid touching other surfaces (e.g., skin, table, etc.)
- After the sample is collected, students should be encouraged to close the tube in a complete manner.
  - Students should be encouraged to tighten the cap until it can no longer turn.
- Close attention should be paid to registering each sample with correct student.

Important Information for Teachers:

Students who are seriously ill should seek immediate medical care from a healthcare professional. Do not wait for results of this test. Call 911 for any medical emergency. Signs of serious medical condition include but are not limited to severe shortness of breath or difficulty breathing, coughing up blood, chest pain, irregular heartbeat, persistent vomiting, or diarrhea.

Ensure that all participating students have parent/guardian consent per school protocol prior to beginning.

Warnings:

* To prevent injury, use only the components provided in the kit to collect the specimen and do not use swab if package seal is broken.
* Do not perform test on an individual under the age of 3.

The Biosearch Technologies SARS-CoV-2 ultra-high-throughput End-Point RT-PCR Test is intended for the qualitative detection of nucleic acid from SARS-CoV-2 in anterior nares specimens.

This Self-collection Kit:

* This product has not been FDA cleared or approved but has been authorized for emergency use by FDA under an EUA.
* This product has been authorized only for the collection and maintenance of nasal swab specimens as an aid in detection of nucleic acid from SARS-CoV-2, not for any other viruses or pathogens.

* The emergency use of this product is only authorized for the duration of the declaration that circumstances exist justifying the authorization of emergency use of medical devices under Section 564(b)(1) of the Federal Food, Drug and Cosmetic Act, 21 U.S.C. § 360bbb-3(b)(1), unless the declaration is terminated, or authorization is revoked sooner.

Getting Started

Before touching supplies and tubes clean your hands with hand sanitizer or soap and water.

Steps in Classroom Administration using Xpress Collect

1. Briefly introduce the activity of self-collecting nose swabs and why it is important as a safety measure sample description is below:

   "*Today we are going to all participate in collecting a sample from our nose that will help to assure we can all meet in class safely. We are going to use this product [hold up Xpress Collect]. It is different from other tests you may have done before. It does not hurt or go all the way up nose.*

   To use this, we first sanitize (or wash) our hands, open the package, swab each side of our nose by rotating it 4x on each side then putting it back into the tube. Once done, I will collect them."

2. Show video of other kids using Xpress Collect and introduce instructions. Sample description is below:

   "*First we will watch a video that will explain the process we'll be using." [show video]*

   "Next I'd like to pass out the instructions" [pass instructions out]

3. Students begin self-collecting

Student Instructions

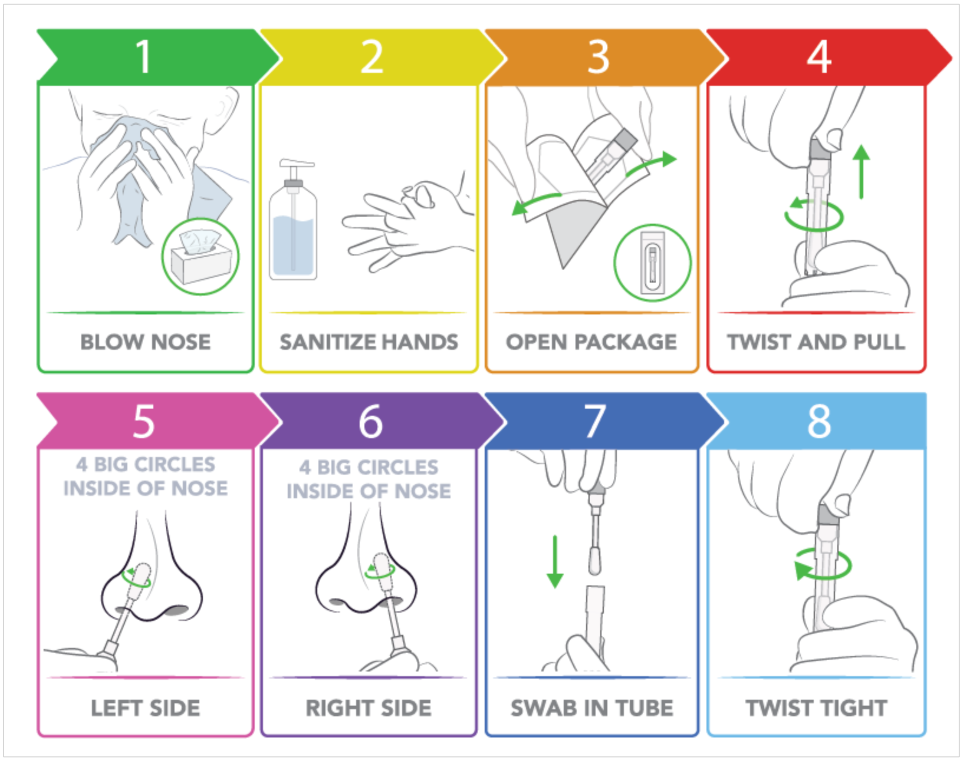

What do I do after my student's samples are collected?

Gather all tubes and transfer to designated school administrator

"Now if each of you could…"

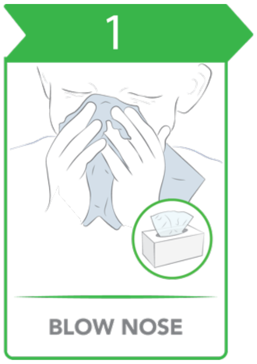

Blow your nose

- A clear nose and clean hands are important to obtain a good sample
- Gently blow nose with a clean tissue to clear both nostrils

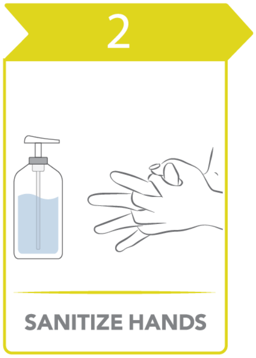

Sanitize your hands

- Clean hands with soap and water or hand sanitizer.
- Ensure hands are dry before proceeding.

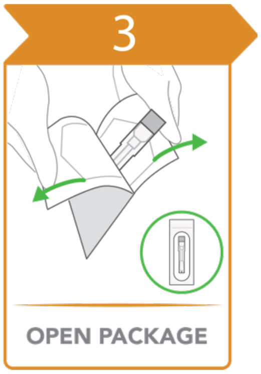

Open the package

- Remove the tube from the wrapper by pulling the two ends of the wrapper apart like a band-aid.

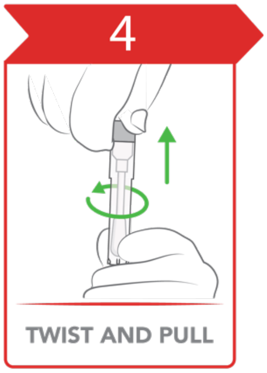

Open the swab and tube

- Twist the cap to remove the swab from the tube. Do not detach the swab from the cap.

⚠ **It is important not to touch the swab tip!**

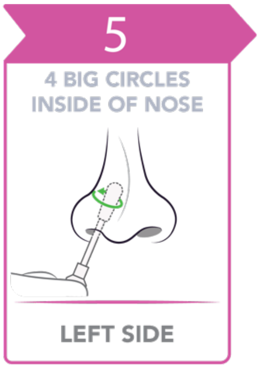

Swab your left nostril

- While holding the cap, gently insert the entire soft tip of the swab into one nostril until you feel a bit of resistance, less than 1/2 an inch
- Using medium pressure, rub the swab slowly in a circular motion around the inside wall of the nostril **four times.**
- The swab tip should be touching the inside wall of the nostril through each rotation.

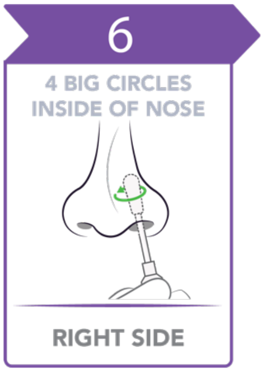

Swab your right nostril

- Students must repeat the same process with the same swab in the other nostril

---

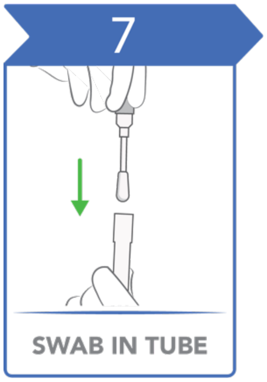

Put the swab back into tube

- While holding the swab cap, put the swab back in the original tube, tip first.

⚠ **It is important not to touch the swab tip!**

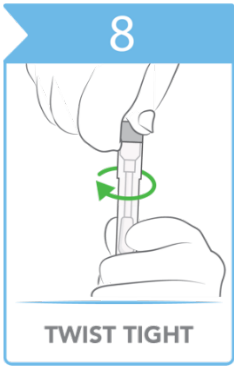

Twist swab tightly into tube

- Screw the swab cap back onto the tube until it cannot go any further. Do not use excessive force to tighten.

Make sure to put your masks back on and sanitize your hands when finished.

Revision History

Revision	Description	Author(s)	Date
0	Initial Release	Laura Haggerty	19-OCT-2021
1	Added note about consent and comparison to other covid tests	Laura Haggerty	21-OCT-2021
2	Modified instructions to include using sanitizer.<br>Updated image of student instructions and teacher step images based on latest revision of student instructions.	Laura Haggerty, Juliana Privitera	26-OCT-2021
3	Modified instructions to match updates to student instructions.<br>Updated images of student instructions.	Juliana Privitera, Kate Abeln	28-OCT-2021
4	Removed the word “home” from page 1	Laura Haggerty	08-NOV-2021
